# N-Myc Regulates Expression of Pluripotency Genes in Neuroblastoma Including *lif, klf2*, *klf4*, and *lin28b*

**DOI:** 10.1371/journal.pone.0005799

**Published:** 2009-06-04

**Authors:** Rebecca Cotterman, Paul S. Knoepfler

**Affiliations:** Department of Cell Biology and Human Anatomy, and Stem Cell Program, University of California Davis School of Medicine, Shriners Hospital For Children Northern California, Sacramento, California, United States of America; City of Hope Medical Center, United States of America

## Abstract

*myc* genes are best known for causing tumors when overexpressed, but recent studies suggest endogenous *myc* regulates pluripotency and self-renewal of stem cells. For example, N-*myc* is associated with a number of tumors including neuroblastoma, but also plays a central role in the function of normal neural stem and precursor cells (NSC). Both c- and N-*myc* also enhance the production of induced pluripotent stem cells (iPSC) and are linked to neural tumor stem cells. The mechanisms by which *myc* regulates normal and neoplastic stem-related functions remain largely open questions. Here from a global, unbiased search for N-Myc bound genes using ChIP-chip assays in neuroblastoma, we found *lif* as a putative N-Myc bound gene with a number of strong N-Myc binding peaks in the promoter region enriched for E-boxes. Amongst putative N-Myc target genes in expression microarray studies in neuroblastoma we also found *lif* and three additional important embryonic stem cell (ESC)-related factors that are linked to production of iPSC: *klf2*, *klf4*, and *lin28b*. To examine the regulation of these genes by N-Myc, we measured their expression using neuroblastoma cells that contain a Tet-regulatable N-*myc* transgene (TET21N) as well as NSC with a nestin-cre driven N-*myc* knockout. N-*myc* levels closely correlated with the expression of all of these genes in neuroblastoma and all but *lif* in NSC. Direct ChIP assays also indicate that N-Myc directly binds the *lif* promoter. N-Myc regulates trimethylation of lysine 4 of histone H3 in the promoter of *lif* and possibly in the promoters of several other stem-related genes. Together these findings indicate that N-Myc regulates overlapping stem-related gene expression programs in neuroblastoma and NSC, supporting a novel model by which amplification of the N-*myc* gene may drive formation of neuroblastoma. They also suggest mechanisms by which Myc proteins more generally contribute to maintenance of pluripotency and self-renewal of ESC as well as to iPSC formation.

## Introduction

*myc* genes encode transcription factors belonging to the basic-helix-loop-helix-zipper (bHLHZ) superfamily. While Myc proteins have homology to other bHLHZ proteins and bind to the classical bHLHZ E-box CACGTG, they appear to be atypical members in a number of ways. They have the ability to regulate both specific gene transcription through discrete chromatin events usually at target gene promoters [1], [2] and maintain very large euchromatic domains [3]. At both specific promoters, but also within much larger chromatin domains Myc is most strongly linked with euchromatic marks including acetylation of lysine 9 (AcK9) and methylation of lysine 4 of histone H3 (triMeK4). Myc is also unusual in that it binds and influences expression of an extremely large number of genes, however most often the influence on expression is surprisingly modest in the range of two-fold [2], [4]–[9].

*myc* is associated with a wide variety of cellular functions including proliferation, apoptosis, cellular metabolism and DNA synthesis (reviewed in [10], [11]). Although *myc* is most well-known for its role in a large variety of human cancers when deregulated, there is growing interest in the normal function of *myc* in stem cells and also *myc* activity in induced pluripotent stem cells (iPSC; reviewed in [12], [13]). *myc* genes are important for the normal functions of a variety of stem and progenitor cells. For example, in mouse ESC (mESC), *myc* plays a central role as an effector of the LIF-STAT pathway [14] and overexpressed *myc* confers LIF-independence to mESC. In the nervous system, disruption of N-*myc* leads to a profound impairment of growth and causes microcephaly as well as retinal defects, phenotypes attributable to perturbance of normal neural stem and progenitor cell (NSC) biology [15]–[17]. c- and N-*myc* are also essential for normal hematopoietic stem cell (HSC) function based on gene knockout (KO) studies [18], [19]. The mid-gestational lethality of constitutive c-*myc* and N-*myc* KO mice [20], [21] may also be caused in part by stem cell defects. The stem cell-related functions of *myc* have in addition been postulated to contribute to formation of tumors, perhaps through transformation of normal stem cells into tumor stem cells. This theory is supported by recent knockdown and knockout studies demonstrating that N-*myc* is essential in neural stem cells and precursors of the cerebellum for medulloblastoma genesis [22], N-*myc* plays a key role in blocking the differentiation of cells of origin of medulloblastoma [23], and c-*myc* is required for glioma stem cell function [24].

Putative Myc bound genes in mESC have been mapped by ChIP-chip [25], [26], but it remains unclear how Myc and its target genes fit into the programs controlling pluripotency and self-renewal as well as how this programming could relate to cancer. In fact, findings conflict on whether Myc operates independently or in conjunction with other stem cell transcription factors [25], [27], [28]. These somewhat conflicting findings suggest a high degree of complexity in how Myc operates in stem cells.

Here we have found that N-Myc regulates the expression of a number of genes encoding stem-related factors in neuroblastoma and in NSC, including *lif*, *klf2*, *klf4*, and *lin28b*. The regulation of *klf2*, *klf4*, and *lin28b* occurs both in tumors and stem cells, suggesting enforced expression of aspects of a pluripotency program by N-Myc may contribute to neuroblastoma formation. N-Myc regulation of endogenous LIF production in neuroblastoma implies a potential role of pluripotency-related growth factor signaling in N-Myc driven neuroblastoma genesis. Together these data suggest Myc regulation of stem-related factors is an important mechanism by which it controls stem cell function and contributes to tumorigenesis.

## Results

### ChIP-chip indicates that N-Myc binds the *lif* promoter in a region containing a canonical Myc E-box, CACGTG

**Figure 1:**
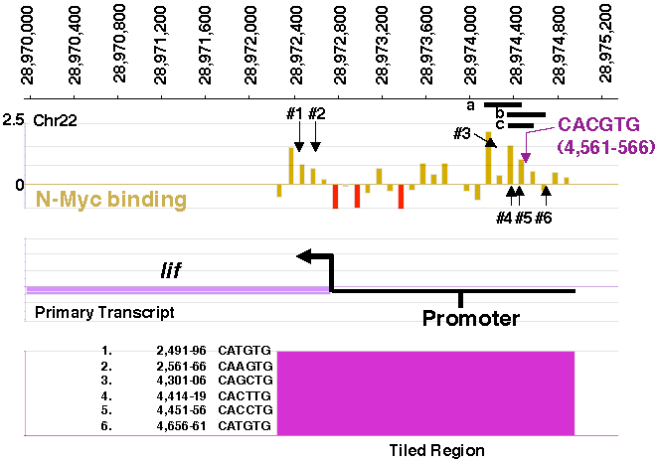
ChIP-chip indicates strong N-Myc binding of the *lif* promoter in human neuroblastoma. ChIP-chip data from previous study [29] was analyzed specifically for *lif* binding by N-*myc*. Several strong peaks were observed (brown). The *lif* promoter is greatly enriched in E-boxes including one canonical *myc* E-box CACGTG shown in purple and 6 non-canonical indicated by downward vertical arrows and listed in tabular form on the bottom left side. The 3 horizontal black bars labeled “a, b, and c” represent the locations of the 3 PCR products from ChIP PCR reactions in Fig. 4.The genomic locations are listed next to the E-boxes and refer to the last 4 digits of the location, with all being in the 28,970,000 base-pair range on chromosome 22.

Previously we used ChIP-chip to analyze N-Myc genomic binding in TET21N neuroblastoma using ENCODE arrays representing 1% of the human genome [29]. By chance the *lif* gene is present on the ENCODE array and we have now conducted further analysis of data from our previous study determining that there are several strong N-Myc binding peaks in the *lif* promoter in TET21N cells (Fig. 1). In the same region as these putative binding peaks is a perfect Myc canonical E-box, CACGTG as well as 6 non-canonical E-boxes.

### Expression microarray and RT-PCR suggest that a number of stem cell related genes are N-Myc targets in neuroblastoma

Because of the evidence from ChIP-chip of direct binding of N-Myc to the *lif* promoter, we conducted further analysis of expression microarray data on human neuroblastoma with a Tet-regulated N-*myc* transgene (TET21N), where we had identified a widespread euchromatic program regulated by N-Myc [29]. We discovered that *lif* expression levels and levels of *klf2*, *klf4*, and *lin28b* from the array studies were sharply decreased in Tet-treated cells (Fig. S2A). Each of the four stem-related genes was downregulated 2–3 fold after 3 and 5 days of Tet-driven repression of N-*myc* expression. Modest re-elevation of N-*myc* due to exhaustion of active Tet in the media at day 7 produced small increases in expression of some of these genes on the microarray. To test the expression microarray findings, conventional RT-PCR was conducted on the same RNA samples used in the previously published study (Fig. S2B). These assays confirmed the general trends of decreased expression of the 4 stem-related genes and indicated re-elevation of expression of the stem-related genes with recovery of N-*myc* levels at day 7 (Figs. S1 and S2B). Thus, N-Myc regulates expression of *klf2*, *klf4*, *lif*, and *lin28b* in human neuroblastoma.

### Quantitative RT-PCR establishes a tight association between N-*myc* expression and that of *klf4*, *klf2*, *lif*, and *lin28b* in neuroblastoma

**Figure 2:**
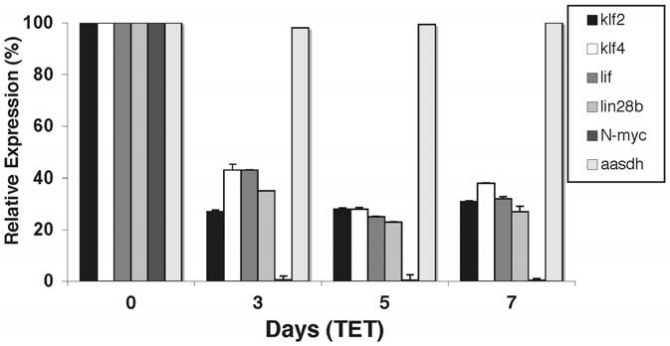
Expression of *klf2*, *klf4*, *lif*, and *lin28b* are linked to N-*myc* levels in human neuroblastoma. qRT-PCR quantifies the changes in *klf2*, *klf4*, *lif*, and *lin28b* expression with similar 3–5 fold decreases in expression for each at each time point of Tet treatment (added daily). Error bars are standard deviation (S.D.) and where not evident it is because the S.D. is so low the bars are so small they do not show up. S.D. throughout this study were quite low and generally ranged from 0.5–5.5%. TET21N neuroblastoma were treated daily with Tet in this experiment. The data here were analyzed using the comparative Ct method, but then reanalyzed using the Pffafl method giving essentially identical results (Fig. S1).

To measure expression changes in *klf2*, *klf4*, *lif*, and *lin28b* in neuroblastoma upon N-*myc* depletion, quantitative RT-PCR (qRT-PCR) was conducted on the cells treated with the same type of 3, 5, and 7-day time-course of Tet treatment that was used for the expression microarray (Fig. 2). There was a clear decrease in expression in the 4 stem genes at each of the 3 time points of Tet treatment with generally 4–5 fold reductions in expression. Unlike in the array experiment where N-*myc* levels modestly recovered at day 7 due to exhaustion of Tet, N-*myc* levels in this subsequent experiment were reduced nearly 200-fold consistently at days 3, 5, and 7. The more robust and sustained decrease in N-*myc* levels in these cells versus those used for the array may explain the somewhat more pronounced reductions in expression of *klf2*, *klf4*, *lif*, and *lin28b*. Together, the expression microarray and both conventional as well as qRT-PCR results indicate that N-*myc* regulates expression of these 4 stem-related genes in TET21N cells.

### Reducing N-*myc* levels rapidly decreases expression of the four stem cell genes and their levels quickly recover upon re-elevation of N-*myc*

**Figure 3:**
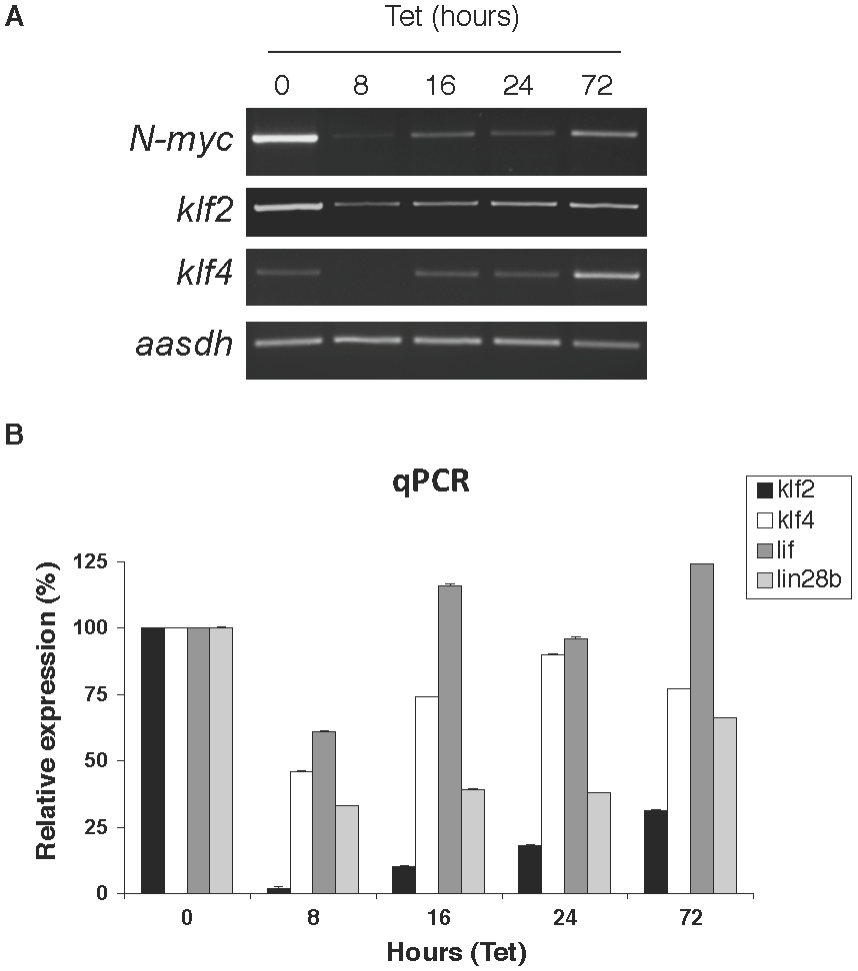
N-*myc* loss rapidly induces loss of expression of *klf2*, *klf4*, *lif*, and *lin28b*. (A) RT-PCR for N-*myc*, *klf2*, and *klf4* was conducted on Tet21N neuroblastoma cells treated for 8, 16, 24, and 72 hours were conducted along with a loading control (*aasdh*, a gene we have found does not vary with modulation of *myc* levels, for example in the TET21N array experiment). N-*myc* levels recovered from 8 hours onward due to a suboptimal dosing of active Tet. (B) qPCR indicates that *klf2*, *klf4*, *lif*, and *lin28b* levels rapidly decreased as after only 8 hours of Tet treatment and consequent short-term loss of N-*myc*. Error bars are S.D.

We next examined the response of the 4 stem cell related genes to a shorter time course of N-*myc* loss and then rapid recovery. Conventional RT-PCR indicated that by 8 hours of Tet treatment, *klf2* and *klf4* were strongly reduced (Fig. 3A). As N-*myc* levels recovered from 16 hours onward, *klf2* and *klf4* gene expression increased largely in parallel. qRT-PCR was used to quantitatively measure changes in *klf2* and *klf4* expression as well as potential changes in expression of *lif* and *lin28b* (Fig 3B). All 4 stem-related genes were decreased by 8 hours of Tet treatment, likely only representing a few hours of loss of N-*myc*. Levels of expression of all 4 stem related genes then recovered over the rest of the time course, tightly linked to the observed recovery of N-*myc* levels.

### N-Myc directly binds the *lif* promoter and regulates triMeK4

**Figure 4:**
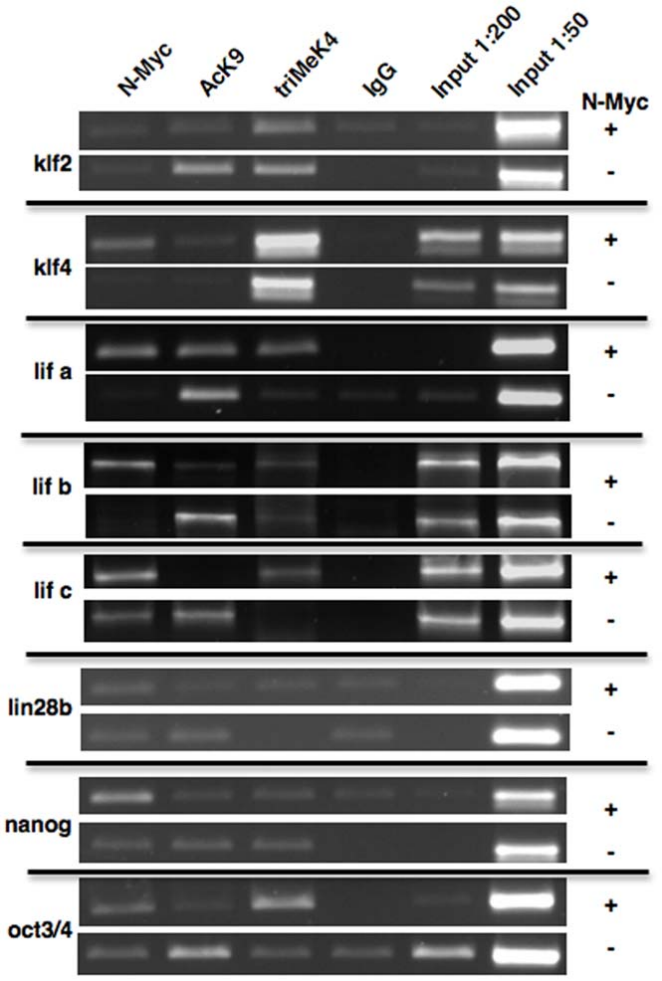
Chromatin immunoprecipitation (ChIP) assays indicate that N-Myc directly binds *lif* and *klf4* in neuroblastoma. ChIP was conducted on Tet21N cells treated daily (3 days) or untreated with Tet. IgG was included as a control along with a 1∶50 and 1∶200 dilutions of input. The ChIP'd *lif* regions a, b, and c are represented by the black bars in Fig. 1. IgG and Input samples were run in parallel as controls.

Transcriptional regulation of gene expression can occur in cascades, particularly when one transcription factor regulates the expression of another, a common phenomenon amongst stem cell-related transcription factors including KLF and Sox2 factors. Thus, it was important to use ChIP to address whether N-Myc was directly binding and regulating expression of *lif*, as suggested by the ChIP-chip studies. Potential direct binding of *klf2*, *klf4*, and *lin28b*, which mostly encode transcription factors that could invoke feedback loops as has been previously reported, was also examined (Fig. 4).

To analyze whether N-Myc directly regulated expression of the stem genes, ChIP was conducted on TET21N cells with or without 3 days of Tet treatment to suppress N-*myc* expression (Fig. 4). Controls in these experiments included the use of IgG as a nonspecific binding control as well as 2 different dilutions of input. Enriched binding of N-Myc was most pronounced for *lif* and *klf4* promoters. This binding was strongly attenuated in the N-*myc* minus cells, also supporting the notion that *lif* and *klf4* are direct targets of N-Myc in neuroblastoma. Both *klf4* and *lif* promoters contain canonical *myc* CACGTG E-boxes in their promoters as well. The *lif* E-box is indicated on the ChIP-chip data in Fig. 1 in purple. Three overlapping but distinct ChIP assays (a, b, and c) were conducted on the *lif* promoter region. All showed enhanced N-Myc binding to the *lif* promoter, but the results for c were less pronounced. The main difference between the b and c regions is that c omits a more 3′ noncanonical E-box, CATGTG. The apparent reduced binding of N-Myc to the c region suggests this E-box may also contribute to N-Myc binding. ChIP results for N-Myc binding to the other pluripotency genes suggested some level of binding but were not conclusive.

Also supporting the notion that *lif* is a direct N-Myc target in neuroblastoma is the significant reduction in triMeK4 in N-*myc* minus cells at its promoter evident by ChIP (Fig 4). Of the 3 *lif* ChIP assays, while a and c showed clear reductions in triMeK4 in N-Myc minus cells, b exhibited only a slight reduction, possibly indicative of differential methylation of histones in this region. Potential modest decreases in triMeK4 were also evident at *lin28b* and *oct3/4*. Interestingly, AcK9 signal was not reduced at any of the genes tested in the N-*myc* minus cells and in fact exhibited consistent increases with loss of N-*myc* at several genes. Together these findings indicate that N-Myc directly regulates *lif* expression at least in part through maintaining triMeK4 in histone H3 associated with the *lif* promoter and may do so at other stem-related genes as well in neuroblastoma.

### N-Myc regulates *klf4*, *klf2*, *lin28b*, and *nanog*, but not *lif* in NSC

**Figure 5:**
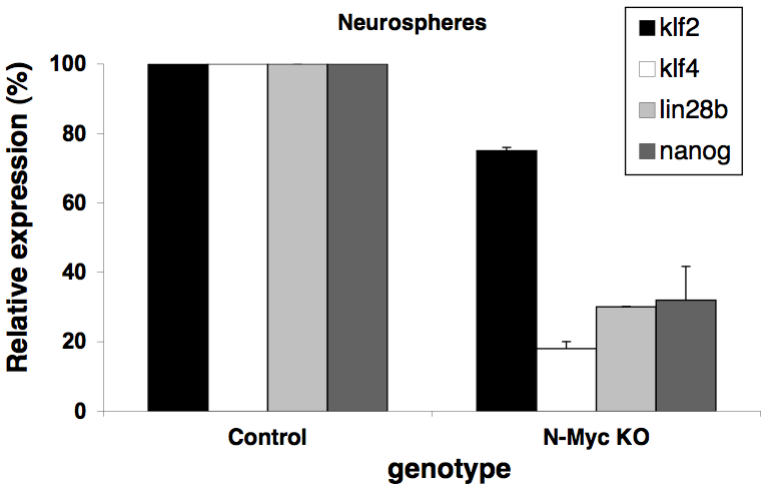
N-Myc regulates *klf2*, *klf4*, and *lin28b* as well as *nanog*, but not *lif* in neurospheres. Control (N-*myc* flox/flox) and N-*myc* null (floxed, nestin-cre) neurospheres were used for qRT-PCR assays. Expression levels in controls were set to 100%. Error bars are S.D.

One possibility is that N-Myc regulation of the stem-related genes is unique to neuroblastoma. Alternatively, N-Myc could regulate their expression in NSC as well as in neuroblastoma. To test these possibilities, RT-PCR was conducted on RNA isolated from control (N-*myc* flox/flox cre negative) and N-*myc* null (N-*myc* flox/flox nestin-cre) murine NSC [15]. *klf2*, *klf4*, and *lin28b* but surprisingly not *lif*, were dependent on N-*myc* for their expression in NSC (data not shown). We next used qRT-PCR to measure expression levels (Fig. 5). *klf4* and *lin28b* were strongly dependent on N-*myc* for their continued expression with reductions in the N-*myc* null NSC at a similar level to that observed in neuroblastoma, while *klf2* was only weakly regulated. To test the possibility that other stem cell genes were regulated in NSC by N-Myc, we conducted RT-PCR for additional stem cells genes and found that *nanog* expression was significantly reduced in N-*myc* null NSC (Fig. 5). Therefore it appears N-Myc regulation of stem-related genes occurs both normally in NSC and in neuroblastoma, however there are some important differences that could play a role in tumorigenesis. For example, *lif* may be regulated specifically in neuroblastoma, while *nanog* appeared only regulated in NSC and not in neuroblastoma.

## Discussion

**Figure 6:**
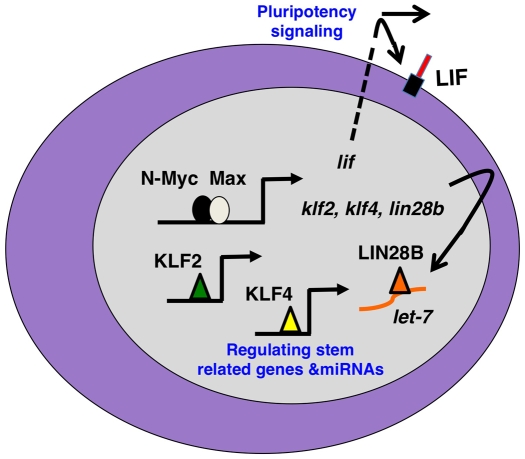
A model of Myc stem-related function in neuroblastoma cells. Two key functions are depicted: growth factor signaling through LIF and induction of stem-related gene expression through induced transcription of genes encoding KLF2, KLF4, and LIN28B. Together these programs are predicted to maintain a “blast”-like state in neuroblastoma tumors through transcriptional and miRNA functions.

The mechanisms by which *myc* regulates both ESC biology and the reprogramming required for iPSC formation are important open questions with critical implications for tumorigenesis as well. Our study suggests a model (Fig. 6) in which *myc* contributes to these pluripotency and self-renewal related functions through inducing expression of pluripotency-related genes including *lif* and those encoding master stem cell factors KLF2, KLF4, and LIN28B. Our findings indicate that a very similar N-Myc regulated program is at work in neuroblastoma and could play a role in its genesis through promoting an aberrant pluripotent state.

Maintaining *lif* expression and expression of *klf2*, *klf4*, and *lin28b* are likely two independent mechanisms by which N-Myc contributes to pluripotency. The regulation of *lif* expression by N-Myc is a mechanism by which it may contribute to neuroblastoma genesis but also ESC and iPSC biology. If N-Myc stimulates the production of *lif* during the early stages of neuroblastoma genesis (Fig. 6), the presence of this potent stem cell related ligand could contribute to tumorigenesis through both autocrine and paracrine signaling that could drive the formation or maintenance of neuroblastoma stem cells (Fig. 6). However, *lif* expression could also be important later in tumorigenesis, perhaps even in tumor maintenance, as a mechanism for preventing differentiation of neuroblastoma. Importantly, our studies were conducted in human neuroblastoma and mouse NSC. While LIF protein has distinct functions in human and mouse ESC, its role in NSC generally is less well understood and there is not currently any evidence of a distinct role for LIF in NSC or neural tumors of different species. However, there is clear evidence that LIF regulates self-renewal and pluripotency of both mouse and human NSC [30], [31].

Of interest is our finding that N-Myc does not appear to regulate *lif* in NSC, suggesting the regulation of *lif* in the neuronal context could be tumor specific. Our discovery of a link between N-Myc and *lif* in neuroblastoma also suggests a possible new treatment for neuroblastoma in the form of LIF antagonists that would be predicted to induce regression through stimulating differentiation (Fig. 6). Besides *lif* expression, we found other differences in stem-related genes regulated by N-Myc in NSC and neuroblastoma. For example, while N-Myc did not appear to be required for *nanog* expression in neuroblastoma, disruption of N-*myc* in NSC caused a pronounced decrease in *nanog* expression. These findings suggest that unique stem cell-related targets exist for N-Myc both in NSC and in neuroblastoma. NSC may be more fully pluripotent, whereas neuroblastoma may express stem-related genes but have at least partially defective pluripotency.

While we have evidence that N-Myc directly regulates *lif* and *klf4* through canonical CACGTG E-boxes and triMeK4 in their promoters, it is also possible that Myc's induction of *lif* is an indirect mechanism by which it acts to also maintain expression of other important pluripotency associated genes that are dependent on the action of LIF as a growth factor. Our findings of N-Myc regulating *lif* in neuroblastoma also fits with previous work indicating overexpression of *myc* confers ectopic LIF-independence on ESC [14]. Together these data suggest that *myc* overexpression in mESC may in part achieve this end through stimulating expression of endogenous LIF. It is important to note that one previous study found that N-*myc* overexpression was correlated with reduced LIF protein levels [32] in some neuroblastoma suggesting that N-*myc* induction of *lif* may occur only in a subset of neuroblastoma. Currently it remains unknown what genes mediate Myc function in mESC to specifically maintain self-renewal and pluripotency, but the targets we have identified here in neuroblastoma are candidates as effectors in ESC as well. Also fitting with our data are the recent observations that c-Myc regulates *lin28* [33] and *lin28b* [34] expression. In the case of *lin28b*, Myc directly binds a canonical CACGTG E-box, suggesting that our findings of N-Myc regulating *lin28b* expression in neuroblastoma and in NSC may be mediated through N-Myc direct binding of this E-box as well. Since LIN28B functions through regulation of miRNA processing including that of let-7, N-Myc activation of LIN28B in neuroblastoma may contribute to maintenance of an miRNA program that enforces an aberrant pluripotent state (Fig. 6).

The regulation of *lif* expression by N-Myc through the CACGTG E-box also correlates with regulation of triMeK4, a key euchromatic histone mark associated with active transcription, within the promoter. Decreased N-Myc expression causes a sharp decrease in triMeK4 accompanied by a pronounced decrease in N-Myc binding. There is also some indication that decreased N-Myc reduces triMeK4 in the promoters of *lin28b* and *oct3/4*. Together these findings suggest N-Myc maintains a transcriptionally active chromatin state at pluripotency genes in neuroblastoma. In contrast, at most of the stem cell-related genes tested, decreased N-Myc surprisingly resulted in increased AcK9 in their promoters. Given the recruitment of histone acetyltransferases by Myc proteins, particularly GCN5 [35] and the dependence on N-Myc for widespread maintenance of AcK9, another key euchromatic mark, in neuroblastoma [29], it is somewhat surprising that decreased N-Myc levels would be accompanied by increased acetylation at these specific genes. At this point it is unclear what mechanism could be responsible for this change and why it would occur specifically at stem cell related genes, but it is possible that pluripotency related genes remain in a poised state rather than be silenced. An increase in AcK9 modification accompanying loss of triMeK4 may prevent a fully silenced state.

Our findings also have implications for iPSC and it is striking that we found N-Myc regulating 3 other known iPSC-related genes, *lin28b*, *klf2*, and *klf4*. These data suggest a model in which overexpressed *myc* enhances iPSC formation in fibroblasts at least in part by turning on *klf* family and *lin28b* gene expression, and through inducing expression of *lif*. Notably our data also provide the first model for why overexpressed *myc*, although a potent enhancer of iPSC formation, may not be formally required for the process: if expression levels of *klf* and *lin28* as well as other pluripotency-related genes are high enough, *myc* may become more dispensable since it is no longer required to turn on their expression. Endogenous *lif* expression may also be dispensable since ectopic LIF is often added to iPSC media. However, alternatively, the ability to generate iPSC without added *myc* may very well be a result of high levels of endogenous *myc* expression. The presence of an iPSC-related gene expression program in neuroblastoma also raises the concern of the tumorigenicity of iPSC.

In our previous model, we proposed that *myc* genes were most likely contributing to tumorigenesis and perhaps iPSC formation through both gene specific and global chromatin events. Our new findings confirm an important role for Myc's gene specific, classical transcription factor function in neuroblastoma. The potential contributions of a more global Myc chromatin function to neuroblastoma genesis and delineating the mechanisms by which Myc contributes to iPSC biology await future study. Particularly important will be functional genomics assays addressing Myc chromatin function, not just binding, in iPSC and in additional types of tumors.

## Materials and Methods

### Cell Culture

All assays were done on exponentially growing Tet21N human neuroblastoma cells [36], either untreated or treated with tetracycline for 3, 5, or 7 at a dose of 1 µg/ml added daily. Tet21N cells do not contain amplified N-*myc* and have little if any endogenous N-*myc* expression [36]. A different preparation of Tet was used in the experiment in Fig. 2 that appeared more potent as it enforced sustained repression of N-*myc* levels throughout that time course.

### Expression microarray studies

RNA samples were prepared from TET21N cells in biological duplicate for expression microarray studies and some of the data from these arrays was previously reported [29]. WG-6 beadchip arrays from Illumina were used. Data was normalized and analyzed using Illumina Beadstudio 3.0 and GeneSpringGX 7.3.1 (Agilent Technologies).

### qRT-PCR

After isolation with the Ambion RNaqueous kit (AM1912, one microgram RNA was treated with DNAse, (Invitrogen, 18068-015), then reverse transcribed with Superscript III First Strand kit (Invitrogen, 18080-040). Samples were diluted to 5 ng/µl, and 25 ng used in qPCR assays. qPCR assays for mouse neurospheres were performed in triplicate using LightCycler 480 Probes Master (Roche, 04 707 494 001) reaction mix with Applied Biosystems Taqman assays on a Roche LightCycler 480.

Applied Biosystems assays are as follows: *klf4* is assay ID Mm00516104-m1, *klf2* is assay ID Mm01244979-g1, *lin28b* is assay ID Mm01190674-m1, *nanog* homeobox assay ID Mm 02019550_s1, and Eukaryotic translation initiation factor 4 gamma 2 (EIF4g2 or Nat1) is assay ID Mm00469036_m1 as a normalizing gene. NOTE: the actual sequences of reagents for these assays are proprietary to Applied Biosystems.

Statistical analysis to calculate the standard deviations for relative expression in different samples was conducted as described in the Applied Biosystems Taqman protocol handbook. Briefly, ΔC_T_ values were obtained and ΔΔC_T_ values (fold differences between reference and testers) were calculated. The standard deviation (SD) of the ΔC_T_ values was calculated as SD = (SD1^2^+SD2^2^)^0.5^. The ΔΔC_T_ value SD is the same as the SD of the ΔC_T_ values since the calibrator value is an arbitrary constant.

Human QPCR assays for Tet21 and Tet B were performed in triplicate using LightCycler 480 SYBR Green I reaction mix (Roche 04 707 516 001) on the same instrument with the following primer sequences:

*klf4* 5′:ACC AGG CAC TAC CGT AAA CAC A

3′: GGT CCG ACC TGG AAA ATG CT

*klf2* 5′:GCG GCA AGA CCT ACA CCA AGA G

3′: GTC CCA GTT GCA GTG GTA GGG

*lin28b* 5′: TGA AAG AAG ACC CAA AGG GAA GAC

3′: TGA TGA TCA AGG CCA CCA CAG T

*lif* 5′:GCA GTG CCA ATG CCC TCT TTA T

3′:CTT GTC CAG GTT GTT GGG GAA C

*nanog* [37] 5′: AAT ACC TCA GCC TCC AGC AGA TG

3′: TGC GTC ACA CCA TTG CTA TTC TTC

(The following two primer sets are from the RTPrimer Database: http://medgen.ugent.be/rtprimerdb/)

N-*myc* 5′:CCG CAA CGA CCT TCG G

3′:TCT TTA CCA ACT CCG GCA CG

c-*myc* 5′: CAA ACC TCC TCA CAG CCC ACT

3′:TTC GCC TCT TGA CAT TCT CCT C

(The primers below are from Primer Bank: http://pga.mgh.harvard.edu/primerbank/)

*aasdh* 5′: TCT GAC CTT CGA TCC TTC TGT

3′:AGA GAA CGC TGG CTA ATT TTG AT

Relative expression levels for SYBR green RTRPCR data were calculated using the algorithm from Pfaffl [38].

### Standard RT PCR

RNA was isolated with the Ambion RNaqueous kit (AM1912). One microgram was treated with DNase I (Invitrogen, 18068-015), reverse transcribed (Invitrogen, 18080-040), and fifty micrograms run in a standard PCR reaction. Primers for mouse and human genes not listed below were from Yamanaka's group [39].

h*klf2* mRNA 1–2 5′: AGC GTG GCT ACA GAG GGT CTC C

3′: CCA AAA ATG CCC ACC TGT CTC T

*hlin28b* mRNA1–2 5′: TCT CAC GAG TTT GGA GCT GAG G

3′:AAT GGC ACT TCT TTG GCT GAG G

*hlif* mRNA 1–2 5′: CCA GAA GAA GAA GCT GGG CTG T

3′: CCT GTG GTC AGG GCT CTT GTA G

*mklf2* mRNA ¾ 5′: GCG TAC ACA CAC AGG TGA GAA G

3′: GTT GGG GAC AGT AAA CTC AAA GG

*mlif* mRNA 1–2 5′: GGC AAC CTC ATCG AAC CAG ATC A

3′: ACC ATC CGA TAC AGC TCC ACC A

*mlin28b* mRNA 3–4 5′: AGG ATG ATT CCA AGA TGC TAC AA

3′: GAG TGC TCT GCC ATT TCT GAC T

### ChIP

Chromatin samples were prepared precisely as previously described [40]. For TET21N cells, 4×15-cm plates (5×10^7^ cells) were crosslinked per experiment. Antibodies used for ChIPs include the following: N-*myc* (2 µg Abcam 16898), AcK9 (2.5 µg 06-942 Upstate, and triMeK4 (5 µl Millipore 04-745). 2 µg Rabbit IgG was used as a background control. Immunoprecipitated chromatin fragments were amplified using the Whole Genome Amplification kit (Sigma) for 2×14 cycles, purified, then checked for enrichment over control IgG and total samples before sending for probing of the ENCODE array (Nimblegen Systems). If inputs between Tet treated (N-Myc -) and untreated (N-Myc +) were substantially different, samples were re-run.

### ChIP Primers

*klf4* promoter 5–6 5′: GTT CGT TCT CTC TGG TCG GGA AA

3′: GTG CGC CGA GTT TGT TGA TTT AG

*klf2* promoter 3–4: 5′: CAA AGC CAC TGG TTC AAG GT

3′: GGG TGA AAT GTG AGC TAA TGT G

*lin28b* 1–2: 5′: GAA TTG TCC AGC AGG GAT TGT

3′: CAA TAC TGC ATT CAT GGT TTG A

*lif* a 5′: AGG TGA TAA AAC TGC CCA TCC

3′: TCT GAG TGG TCA GGT CCT TGT

*lif* b 5′: CAT CTC CTG CAC AAG GAC CTG AC

3′: GGT GGA TTA TAG GGC TGA TGT GG

*lif* c 5′: CAT CTC CTG CAC AAG GAC CTG AC

3′: TGG GCA GAA TGG TAG ATG TAG GG

*nanog* promoter 1–2 5′: CCC AGC CCA GTT AAT TTT TGT

3′: TGT CCC ATT GTG TCT AGG GTA A

*oct3/4* promoter 1–2 5′: CAG TAT CGG GAT GGG AAT G

3′: CAC CAC ACC CAA CTT TCA AC

## Supporting Information

Figure S1 (0.06 MB DOC)

Figure S2 (0.08 MB DOC)
