# Supplementary figures and images for: N-Myc Regulates Expression of Pluripotency Genes in Neuroblastoma Including lif, klf2, klf4, and lin28b
Source: PLoS One. 2009 Jun 4;4(6):e5799. doi: 10.1371/journal.pone.0005799 (PMC2686170; doi:10.1371/journal.pone.0005799)

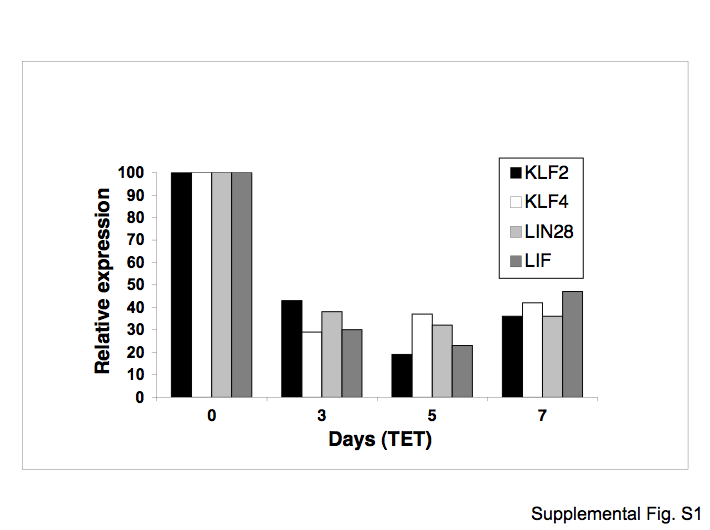


**Fig. S1. qRTPCR data analyzed using the Pffafl method strongly parallel the results in Fig. 2.**

Supplement: Figure S1 — (0.06 MB DOC) [file pone.0005799.s001.doc]
