# Supplementary material for: N-Myc Regulates Expression of Pluripotency Genes in Neuroblastoma Including lif, klf2, klf4, and lin28b
Source: PLoS One. 2009 Jun 4;4(6):e5799. doi: 10.1371/journal.pone.0005799 (PMC2686170; doi:10.1371/journal.pone.0005799)
Supplement: Figure S2 — (0.08 MB DOC) [file pone.0005799.s002.doc]

**
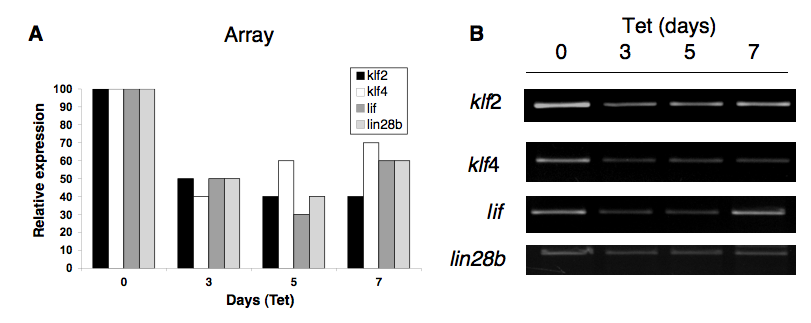
**

**Fig. S2. (A) Expression microarray on neuroblastoma cells with a Tet-repressible N-*myc* transgene for 3, 5, and 7 days indicate that decreasing N-*myc* consistently results in lower expression of *klf2*, *klf4*, *lif*, and *lin28b*.** Arrays were conducted in biological duplicate for each time point and data are the mean expression from both samples. (B) RTPCR on the same RNA samples used for the array validates the gene expression changes seen in the array.
